# Exenatide Facilitates Recovery from Oxaliplatin-Induced Peripheral Neuropathy in Rats

**DOI:** 10.1371/journal.pone.0141921

**Published:** 2015-11-04

**Authors:** Shunsuke Fujita, Soichiro Ushio, Nana Ozawa, Ken Masuguchi, Takehiro Kawashiri, Ryozo Oishi, Nobuaki Egashira

**Affiliations:** Department of Pharmacy, Kyushu University Hospital, Fukuoka, Japan; University of Lancaster, UNITED KINGDOM

## Abstract

**Background:**

Oxaliplatin has widely been used as a key drug in the treatment of colorectal cancer; however, it causes peripheral neuropathy. Exenatide, a glucagon-like peptide-1 (GLP-1) agonist, is an incretin mimetic secreted from ileal L cells, which is clinically used to treat type 2 diabetes mellitus. GLP-1 receptor agonists have been reported to exhibit neuroprotective effects on the central and peripheral nervous systems. In this study, we investigated the effects of exenatide on oxaliplatin-induced neuropathy in rats and cultured cells.

**Methods:**

Oxaliplatin (4 mg/kg) was administered intravenously twice per week for 4 weeks, and mechanical allodynia was evaluated using the von Frey test in rats. Axonal degeneration was assessed by toluidine blue staining of sciatic nerves.

**Results:**

Repeated administration of oxaliplatin caused mechanical allodynia from day 14 to 49. Although the co-administration of extended-release exenatide (100 μg/kg) could not inhibit the incidence of oxaliplatin-induced mechanical allodynia, it facilitated recovery from the oxaliplatin-induced neuropathy with reparation of axonal degeneration. Inhibition of neurite outgrowth was evaluated in cultured pheochromocytoma 12 (PC12) cells. Exenatide inhibited oxaliplatin-induced neurite degeneration, but did not affect oxaliplatin-induced cell injury in cultured PC12 cells. Additionally, extended-release exenatide had no effect on the anti-tumor activity of oxaliplatin in cultured murine colon adenocarcinoma 26 (C-26) cells or C-26 cell-implanted mice.

**Conclusion:**

These results suggest that exenatide may be useful for treating peripheral neuropathy induced by oxaliplatin in colorectal cancer patients with type 2 diabetes.

## Introduction

Oxaliplatin, a platinum-based chemotherapeutic agent, is a key drug widely used for treating colorectal cancer; however, it causes severe acute and chronic peripheral neuropathies. In the early phase after administration, the acute neuropathy occurs in 85 to 95% of all patients and they suffer from cold hyperesthesia [1]. It has been thought that the acute neuropathy is not due to morphological damage of the nerve [2] and is due to alternations of voltage-gated Ca^2+^ and K^+^ channels [3, 4]. Recently, transient receptor potential (TRP) melastatin (M) 8 and ankyrin (A) 1 channels have been reported to play an important role in the oxaliplatin-induced acute cold hypersensitivity in rodents [5, 6]. More recently, the voltage-gated Nav1.9 sodium channel has been reported to be associated with cold pain hypersensitivity induced by oxaliplatin [7]. After multiple cycles, patients develop chronic neuropathy, which is characterized by sensory and motor dysfunction [8]. This chronic neuropathy is dose limiting and often not only limits the patient's activities of daily living, but also hinders long-term use of oxaliplatin-based chemotherapy [9]. Moreover, the chronic neuropathy takes at least 2 years after discontinuation of the drug to completely recover from the neuropathy in 10% of patients [10]. Thus, these neuropathies are a major clinical problem in oxaliplatin chemotherapy.

Oxaliplatin causes damage to cell bodies [11–13] and selective atrophy of subpopulations of dorsal root ganglion (DRG) neurons [14]. Recently, the activation of spinal astrocytes has been reported to be involved in the oxaliplatin-induced neuropathic pain [15–17]. *N*-Palmitoylethanolamine, the endogenous amide belongs to the family of fatty acid ethanolamides (FAEs), has been reported to modulate glial cells and exert antinociceptive effects on oxaliplatin-induced neuropathic pain in rats [18]. Furthermore, it has been reported that α7 nicotinic acetylcholine receptor (nAChR) subtype is involved in the oxaliplatin-induced neuropathy, and α7 nAChR-dependent modulation of glial functions improves the pain-related behavior [19]. Oxaliplatin is metabolized to oxalate and dichloro (1,2-diaminocyclohexane) platinum [Pt(dach)Cl_2_] [20]. We previously reported that repeated administration of oxaliplatin induced cold allodynia from day 5 and mechanical allodynia from day 15 in rats [21]. Oxaliplatin causes degeneration of myelinated fibers in the rat sciatic nerve and inhibits neurite outgrowth in cultured pheochromocytoma 12 (PC12) and rat DRG cells [21]. We also demonstrated the involvement of Pt(dach)Cl_2_ in mechanical allodynia but not cold allodynia [22]. Moreover, we showed that this mechanical allodynia is characterized by increased expression of the NR2B-containing *N*-methyl-D-aspartate (NMDA) receptor and enhanced calcium/calmodulin-dependent protein kinase II (CaMKII) phosphorylation in the spinal cord [23, 24]. Most recently, we indicated that oxaliplatin induces hypomyelination and reduces expression of neuregulin 1, a myelination regulatory factor, in the rat sciatic nerve [25]. However, the mechanisms of oxaliplatin-induced peripheral neuropathy have yet to be elucidated fully and preventive strategies have not yet been established [26].

Glucagon-like peptide-1 (GLP-1) is secreted from ileal L cells and stimulates the pancreas to secrete insulin via GLP-1 receptors. Exenatide is a GLP-1 receptor agonist that is clinically used to treat type 2 diabetes. Recently, GLP-1 receptor agonists have been shown to prevent or improve diabetic neuropathy model [27–29], and nerve crush and pyridoxine-induced neuropathy models [30, 31]. GLP-1 receptor agonist has also been reported to prevent against mitochondrial apoptotic pathway induced by spinal cord injury in rats [32]. Moreover, GLP-1 and GLP-1 receptor agonists have been reported to show neurotrophic and neuroprotective properties [33–35]. However, the effects of GLP-1 receptor agonists on oxaliplatin-induced peripheral neuropathy have not been studied. In the present study, we examined the effects of exenatide on oxaliplatin-induced inhibition of neurite outgrowth in cultured PC12 cells and oxaliplatin-induced mechanical allodynia in rats.

## Materials and Methods

### Animals

Six-week-old male Sprague-Dawley rats weighing 200–250 g (Kyudo Co., Tosu, Japan) were used in the present study. Six-week-old male BALB/c mice weighing 21–23 g (CLEA Japan, Inc., Tokyo, Japan) were used for the *in vivo* tumor growth model. Animals were housed in groups of three to four per cage, with lights on from 07:00–19:00 h. Animals had free access to food and water in their home cages. All experiments were approved by the Experimental Animal Care and Use Committee of Kyushu University according to the National Institutes of Health guidelines (Permit Number: A27-011-0), and followed the International Association for the Study of Pain Committee for Research and Ethical Issues guidelines for animal research [36].

### Cell cultures

PC12 cells and murine colon adenocarcinoma 26 (C-26) cells were obtained from Riken (Saitama, Japan). Cells were maintained in DMEM (MP Biomedicals Inc., Irvine, CA, USA) containing 2 mM L-glutamine, 10% (v/v) fetal bovine serum and 10% (v/v) horse serum in a humidified atmosphere containing 5% (v/v) CO_2_ at 37°C.

### Drugs

Oxaliplatin (Elplat^®^) was obtained from Yakult Co., Ltd. (Tokyo, Japan) and was dissolved in 5% (w/v) glucose solution. Exenatide (Byetta^®^) and extended-release exenatide (Bydureon^®^) were purchased from AstraZeneca, Inc. (London, UK) and were used for *in vitro* and *in vivo* studies, respectively. Oxaliplatin (4 mg/kg) or vehicle (5% (w/v) glucose solution) was injected intravenously (i.v.), twice per week for 4 weeks (days 1, 2, 8, 9, 15, 16, 22, and 23). Oxaliplatin was administrated at a volume of 1 mL/kg of body weight. Extended-release exenatide (100 μg/kg) was injected subcutaneously (s.c.), once per week for 9 weeks (days 1, 8, 15, 22, 29, 36, 43, 50, and 57). The doses of oxaliplatin and exenatide were chosen based on previous reports [21, 37].

### Cell viability assay of cultured PC12 and C-26 cells

PC12 and C-26 cells were seeded at a density of 6 × 10^4^ cells/well in 24-well plates and were used for experiments on the following day. PC12 cells were incubated in serum-free medium with nerve growth factor-b, rat recombinant carrier free (NGF; 60 μM; Wako Pure Chemicals, Osaka, Japan) for 24 h followed by exposure to oxaliplatin (3 μM) and exenatide (3, 10, or 30 nM) for 24 h. C-26 cells were incubated in serum-free medium and exposed to oxaliplatin (70 μM) and exenatide for 24 h. The concentrations of these drugs were chosen based on previous reports [21, 38, 39]. In addition, the concentration of oxaliplatin in C-26 cells was determined by the preliminary study. This concentration (70 μM) is required to reduce tumor growth by about 30%. Cell viability was assessed by the reduction of 2-(2-methoxy-4-nitrophenyl)-3-(4-nitrophenyl)-5-(2,4-disulfophenyl)-2H-tetrazolium monosodium salt (WST-8) to formazan by mitochondria. Briefly, after treatment with oxaliplatin and exenatide, cells were washed with phosphate-buffered saline (PBS). Cells were incubated with 210 μL serum-free medium and 10 μL of WST-8 assay solution (Cell Counting Kit-8; Dojindo Laboratory, Kumamoto, Japan) for 1 h at 37°C in humidified air supplemented with 5% (v/v) CO_2_. The incubation medium was carefully removed and transferred to 96-well flat-bottom plastic plates (Nalge Nunc International, Rochester, NY, USA). The amount of formazan formed was measured from the absorbance at 450 nm with a reference wavelength of 620 nm using a microplate reader (Sunrise; TECAN Group Ltd., Mannedorf, Switzerland).

### Assessment of PC12 neurite outgrowth

PC12 cells differentiate in the presence of NGF, and have previously been used as a model of oxaliplatin-induced neurodegeneration *in vitro* [21]. PC12 cells were seeded at a density of 5 × 10^3^ cells/cm^2^ in 96-well plates (Falcon; Becton Dickinson Co., Ltd., NJ, USA) and were used for experiments on the third day. Cultured cells were exposed to oxaliplatin (3 μM) and exenatide (3, 10, or 30 nM) for 24 h. They were stained with Calcein-AM (1:1000, Dojindo Laboratory, Kumamoto, Japan) and Hoechst 33342 (1:1000, Dojindo Laboratory) at 37°C for 10 min. Cells were monitored using ImageXpress (Molecular Devices Japan, Tokyo, Japan) and the lengths of neurite (number of neurite: from 600 to 1000) were measured using MetaXpress (Molecular Devices Japan) three times repeatedly.

### von Frey testing for mechanical allodynia

We investigated the effect of exenatide on mechanical allodynia using the von Frey test. This test was performed before the first drug administration (day 0) and on days 7, 14, 21, 28, 35, 42, 49, 56, and 63. Rats were placed in a clear plastic box (20 × 17 × 13 cm) with a wire mesh floor and allowed to habituate for 30 min prior to testing. Filaments (The Touch Test Sensory Evaluator Set; Linton Instrumentation, Norfolk, UK) in the range of 1–15 g bending force were applied to the mid-plantar skin of each hind paw six times, with each application held for 6 s. The paw withdrawal threshold was determined by a modified up-down method as previously described by Kawashiri et al. [21].

### Assessment of sciatic axonal degeneration

On days 28, 42, and 63, sciatic nerves were harvested from rats anesthetized with sodium pentobarbital. Nerves were fixed in 2% (w/v) glutaraldehyde in 0.1 M phosphate buffer (pH 7.4, 4°C) for 4 h followed by washing with 0.1 M phosphate buffer. After 8% (w/v) sucrose-substitution, each sample was cut into 5 mm length and embedded in paraffin. Paraffin blocks were sliced into 3 μm section, and each section was stained with 0.05% toluidine blue. Four sections were examined per mouse. Sample sections were evaluated using light microscopy (BX51; Olympus Corp., Tokyo, Japan). Axon area was calculated by image analysis software (Image J 1.36; Wayne Rasband, National Institutes of Health, MD, USA) from approximately 3000 to 6000 axons per group.

### Tumor growth analysis

C-26 cells (1.0 × 10^6^ cells per mouse in 50 μL PBS) were implanted subcutaneously in the right paw of BALB/c mice. Six days after implantation of tumor cells, administration of drugs was started. Oxaliplatin (6 mg/kg, i.p. days 1, 2, 8, and 9) and extended-release exenatide (150 μg/kg, s.c. days 1 and 8) were injected respectively. The dose of oxaliplatin followed previous report [40], and the dose of exenatide was chosen based on a previous report [41]. The tumor volumes were measured on days 0, 4, 7, and 11, and calculated as follows: Volume (mm^3^) = π/6 × Thickness (mm) × Length (mm) × Width (mm).

### Statistical analyses

Values are expressed as the mean ± standard error mean. The values were analyzed by the Student’s *t*-test, or one-way analysis of variance followed by the Tukey-Kramer test (StatView; Abacus Concepts, Berkely, CA, USA) to determine differences among the groups. Cell viability data of cultured PC12 and C-26 cells and neurite outgrowth measurements of PC12 cells are expressed as percentages of the vehicle-treated group. A *P* < 0.05 was accepted as statistically significant.

## Results

### Effect of exenatide on oxaliplatin-induced neurite degeneration in cultured PC12 cells

**Fig 1:**
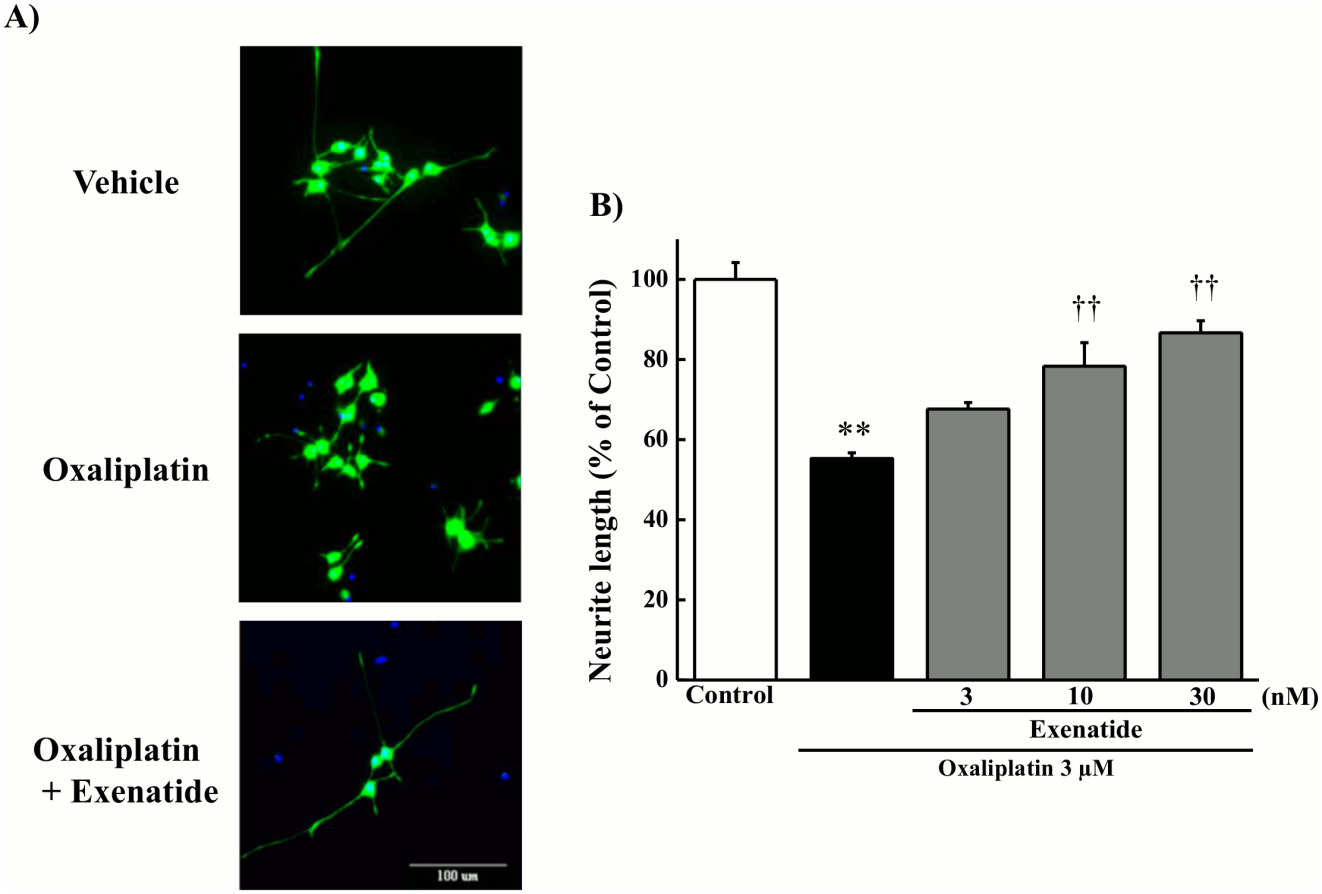
Effect of exenatide on oxaliplatin-induced neurite degeneration in cultured PC12 cells. Cultured PC12 cells were incubated with oxaliplatin (3 μM) for 24 h in the presence or absence of exenatide (3, 10, or 30 nM). (A) Photographs were originally magnified 800 ×. Scale bar = 100 μm. (B) Neurite length was measured using image analysis software (MetaXpress). Results are expressed as the mean ± standard error mean (*n* = 3). **P < 0.01 compared with control, ††*P* < 0.01 compared with oxaliplatin alone.

Exposure to oxaliplatin (3 μM) for 24 h shortened the length of PC12 neurites (Fig 1). The exposure to exenatide (10 and 30 nM) significantly prevented the oxaliplatin-induced inhibition of neurite outgrowth (10 and 30 nM, *P* < 0.01 by the Tukey-Kramer test).

### Effect of exenatide on oxaliplatin-induced cell viability in cultured PC12 cells

**Fig 2:**
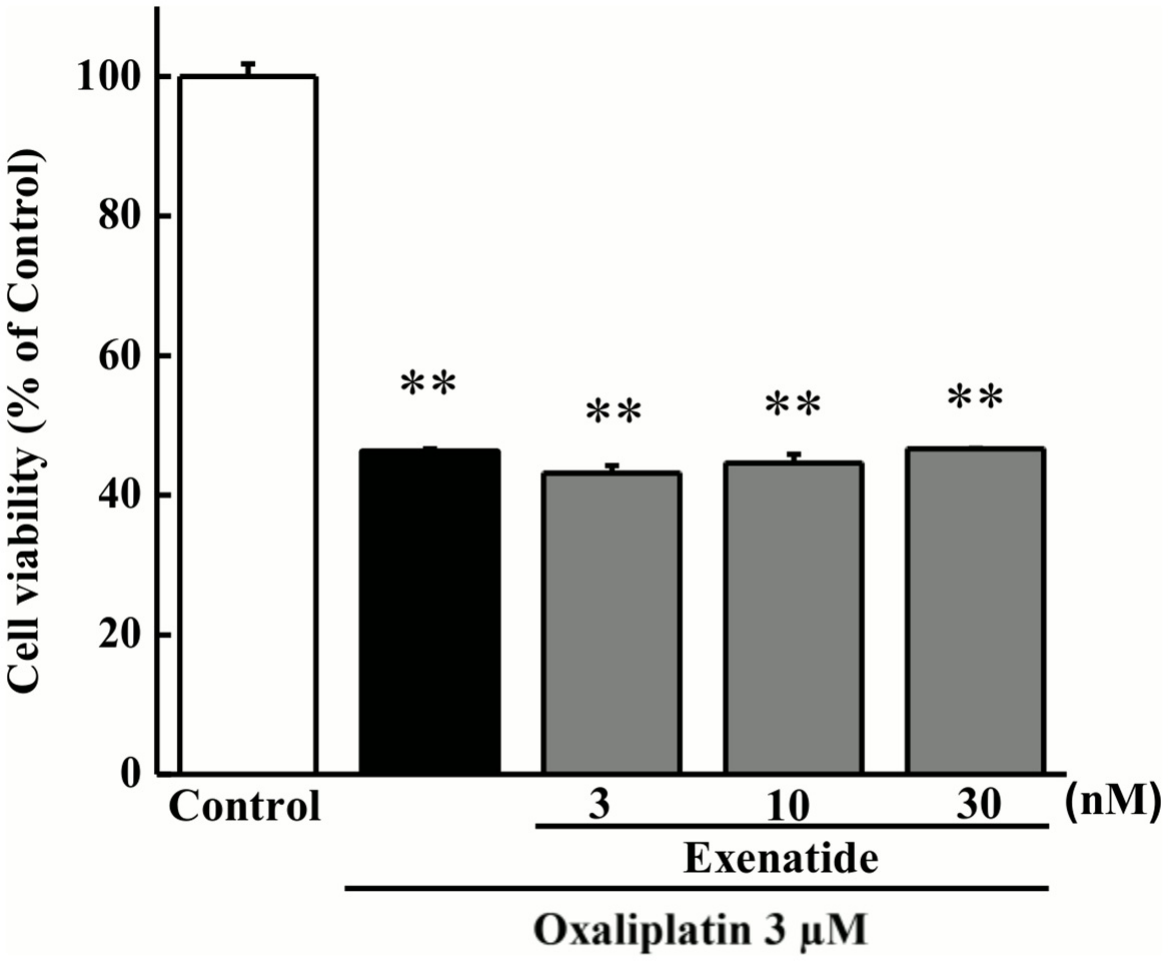
Effect of exenatide on oxaliplatin-induced cell viability in cultured PC12 cells. Cultured PC12 cells were incubated with oxaliplatin (3 μM) for 24 h in the presence or absence of exenatide (3, 10, or 30 nM). Cell viability was measured using the WST-8 assay. Results are expressed as the mean ± standard error mean (*n* = 4). **P < 0.01 compared with control.

As shown in Fig 2, exposure to oxaliplatin (3 μM) for 24 h decreased cell viability in cultured PC12 cells (*P* < 0.01 by the Tukey-Kramer test). Co-exposure to exenatide (3, 10, or 30 nM) for 24 h had no effect on the oxaliplatin-induced decrease in cell viability.

### Effects of extended-release exenatide on incidence of mechanical allodynia and histological changes induced by oxaliplatin

**Fig 3:**
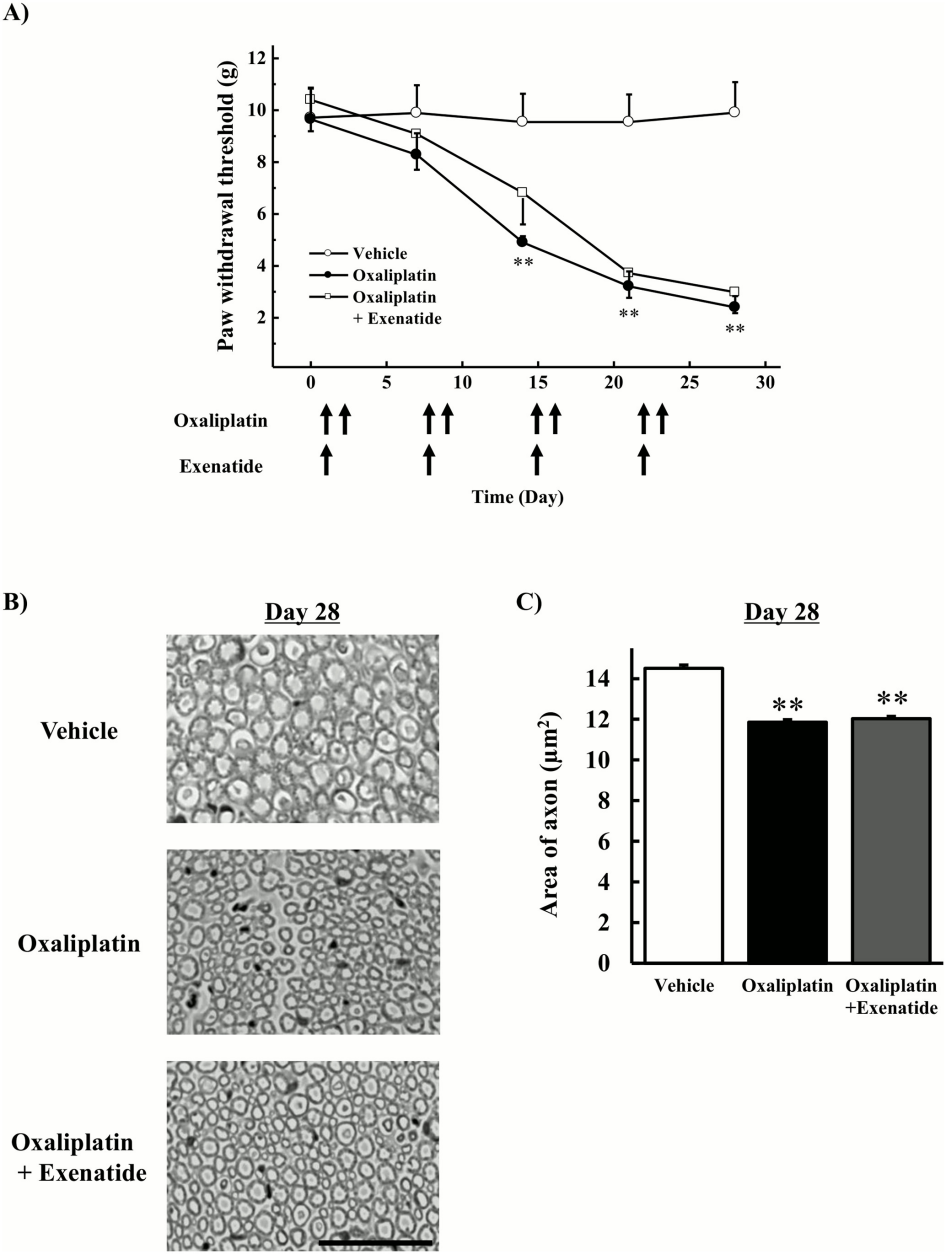
Effects of extended-release exenatide on incidence of mechanical allodynia and axonal degeneration induced by oxaliplatin. Oxaliplatin (4 mg/kg) was administered i.v. twice per week for 4 weeks (days 1, 2, 8, 9, 15, 16, 22, and 23). Extended-release exenatide (100 μg/kg) was administered s.c. once per week for 4 weeks. (A) The von Frey test was performed before the first drug administration (on day 0) and on days 7, 14, 21, and 28. Values are expressed as the mean ± standard error mean of five to six animals. (B) On day 28, the sciatic nerve was harvested, and samples were stained with toluidine blue. Images were captured at 800× magnification. Scale bar = 60 μm. (C) The area of axon was calculated by image analysis software (Image J 1.36) from approximately 3000 to 6000 axons per group. Values are expressed as the mean ± standard error mean of four animals. **P < 0.01 compared with vehicle.

Animals were injected twice weekly with oxaliplatin for 4 weeks and once weekly with extended-release exenatide for 4 weeks. Oxaliplatin (4 mg/kg, i.v., twice per week) significantly lowered the withdrawal threshold compared with the vehicle on days 14, 21, and 28 (*P* < 0.01 by the Tukey-Kramer test, Fig 3A). Co-administration of extended-release exenatide (100 μg/kg, s.c.) had no effect on the oxaliplatin-induced reduction in withdrawal threshold. Histological studies revealed atrophy of myelinated fibers in the rat sciatic nerve of oxaliplatin-treated animals and rats co-administered oxaliplatin and exenatide for 4 weeks (Fig 3B). The quantification analysis showed that oxaliplatin significantly decreased area of axon (*P* < 0.01 by the Tukey-Kramer test, Fig 3C), and co-treatment with exenatide had no effect on the oxaliplatin-induced decrease in area of axon.

### Effects of extended-release exenatide on recovery from mechanical allodynia and histological changes induced by oxaliplatin

**Fig 4:**
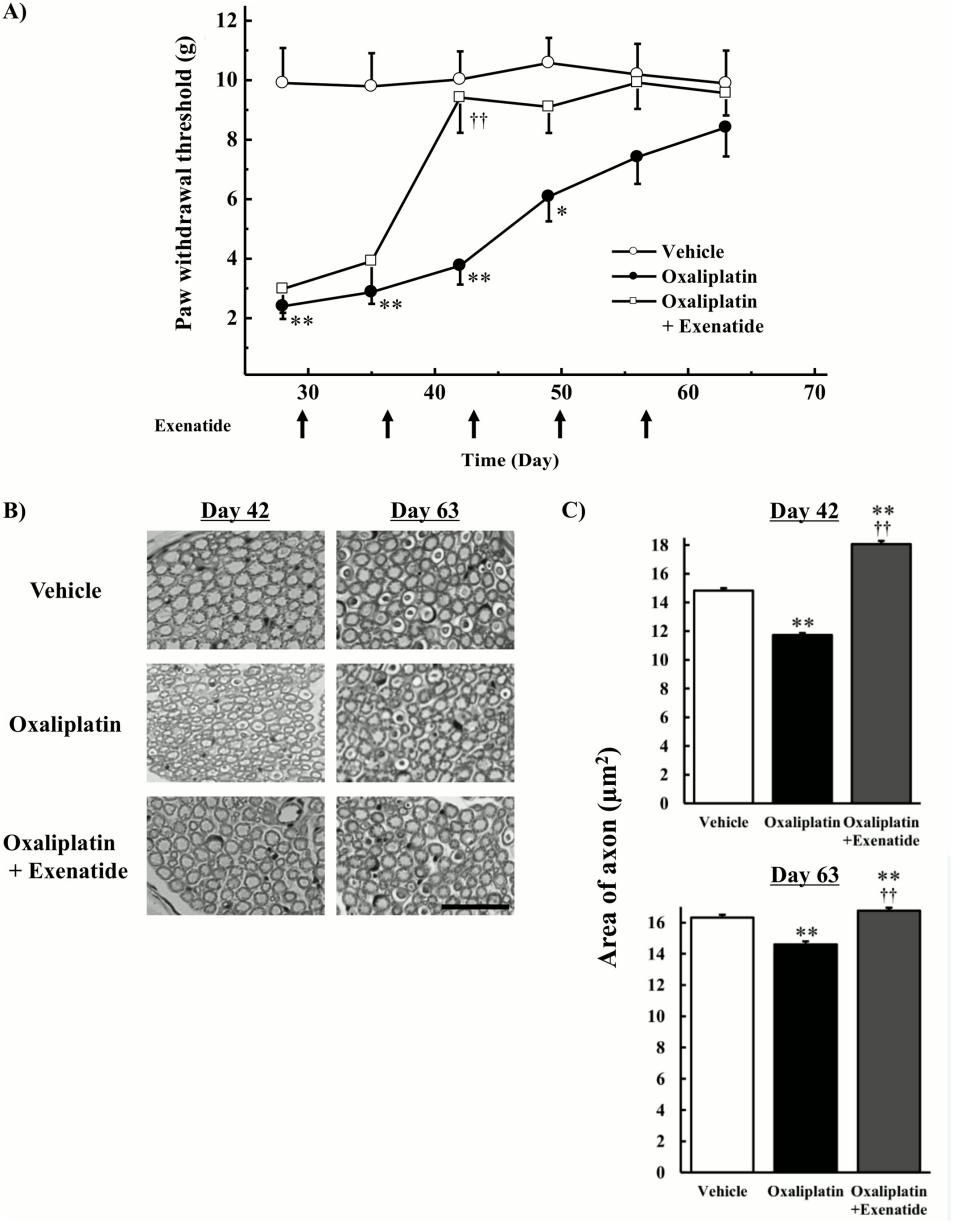
Effects of extended-release exenatide on recovery from mechanical allodynia and axonal degeneration induced by oxaliplatin. Oxaliplatin (4 mg/kg) was administered i.v. twice per week for 4 weeks (days 1, 2, 8, 9, 15, 16, 22, and 23). Extended-release exenatide (100 μg/kg) was administered s.c. once per week for 9 weeks (days 1, 8, 15, 22, 29, 36, 43, 50, and 57). Oxaliplatin treatment ceased on day 23, however exenatide treatment was continued for an additional 5 weeks. (A) The von Frey test was performed before the first drug administration (on day 0) and on days 35, 42, 49, 56, and 63. Values are expressed as the mean ± standard error mean of five to six animals. (B) On days 42 and 63, the sciatic nerve was harvested, and samples were stained with toluidine blue. Images were captured at 800× magnification. Scale bar = 60 μm. (C) The area of axon (number of axon: from 3000 to 6000/field) was measured using image analysis software (Image J 1.36). Values are expressed as the mean ± standard error mean of four animals. *P < 0.05, **P < 0.01 compared with vehicle, ††*P* < 0.01 compared with oxaliplatin alone.

Animals were injected twice weekly with oxaliplatin for 4 weeks and once weekly with extended-release exenatide for 9 weeks. Oxaliplatin treatment ceased on day 23, however exenatide treatment was continued for an additional 5 weeks. Co-administration of extended-release exenatide (100 μg/kg, s.c.) significantly relieved oxaliplatin-induced mechanical allodynia on day 42 (*P* < 0.01 by the Tukey-Kramer test, Fig 4A). On day 63, no behavioral abnormalities were observed in rats treated with oxaliplatin. On day 42, atrophy of the sciatic nerve was observed in oxaliplatin-treated rats, but no histological abnormalities were observed in the co-treatment with exenatide group (Fig 4B). The quantification analysis showed that oxaliplatin significantly decreased area of axon (*P* < 0.01 by the Tukey-Kramer test), and co-treatment with exenatide reversed the oxaliplatin-induced decrease in area of axon (*P* < 0.01 by the Tukey-Kramer test, Fig 4C). On day 63, histological changes were little observed in rats treated with oxaliplatin (Fig 4B), but the quantification analysis showed that co-treatment with exenatide reversed the oxaliplatin-induced decrease in area of axon (*P* < 0.01 by the Tukey-Kramer test, Fig 4C).

### Effect of exenatide on oxaliplatin-induced tumor cytotoxicity

**Fig 5:**
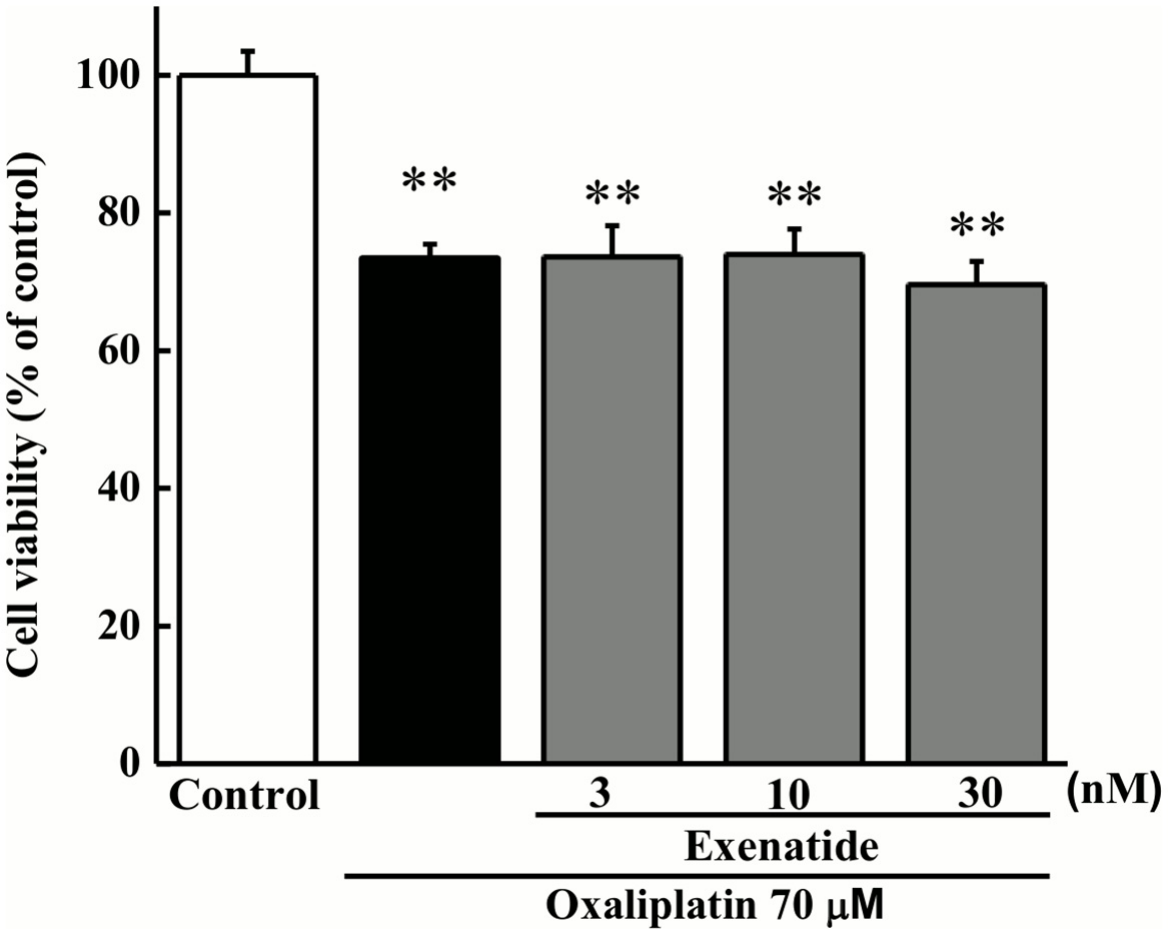
Effect of exenatide on oxaliplatin-induced tumor cytotoxicity. C-26 cells were incubated with oxaliplatin (70 μM) for 24 h in the presence or absence of exenatide (3, 10, or 30 μg/mL). Cell viability was measured using the WST-8 assay. Values are expressed as a percentage of the control (*n* = 4). **P < 0.01 compared with control.

Exposure of cultured C-26 cells to oxaliplatin (70 μM) for 24 h significantly decreased tumor cell viability (*P* < 0.01 by the Tukey-Kramer test, Fig 5). Exenatide (3, 10 or 30 nM) had no effect on the oxaliplatin-induced decrease in cell viability.

### Effect of extended-release exenatide on the anti-tumor activity of oxaliplatin in tumor cell-implanted mice

**Fig 6:**
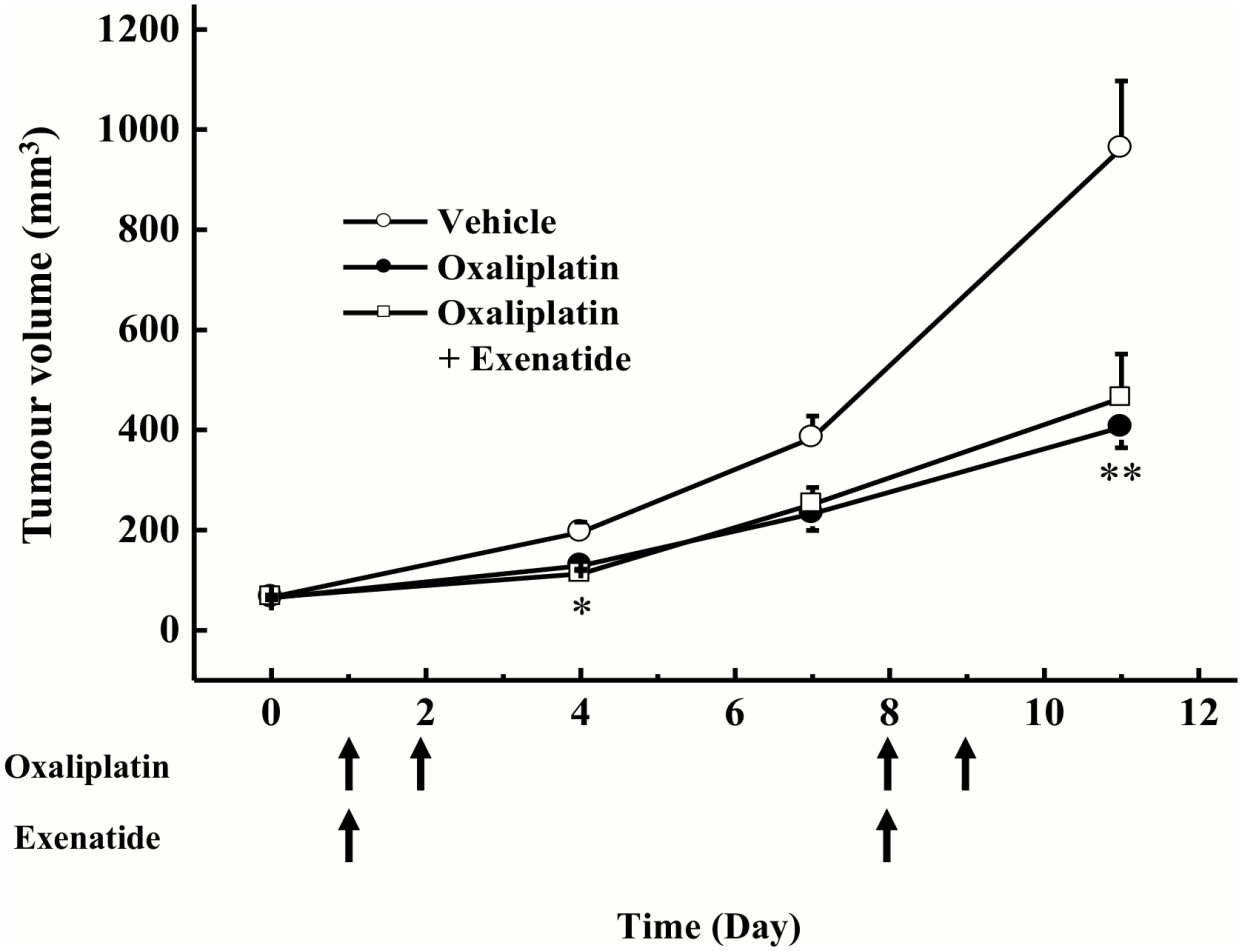
Effect of extended-release exenatide on oxaliplatin-induced anti-tumor activity in tumor cell-implanted mice. C-26 cell-implanted mice were treated with oxaliplatin (6 mg/kg, i.p. days 1, 2, 8, and 9) and extended-release exenatide (150 μg/kg, s.c. days 1 and 8). Tumor volumes were measured on days 0, 4, 7, and 11. Values are expressed as the mean ± standard error mean of 8–12 animals on days 0, 4, 7, and 11. *P < 0.05, **P < 0.01 compared with vehicle.

Oxaliplatin (6 mg/kg, i.p.) significantly inhibited the increase in tumor volume when compared with vehicle in tumor cell-implanted mice (day 4: *P* < 0.05, day 11: *P* < 0.01 by Tukey-Kramer test, Fig 6). Extended-release exenatide (150 μg/kg, s.c.) had no effect on the oxaliplatin-induced inhibition of tumor growth.

## Discussion

In the present study, oxaliplatin caused mechanical allodynia from day 14, which gradually dissipated after the end of oxaliplatin administration, and these results are consistent with our previous report [21]. Clinically, it has been reported to take 2 or more years to completely recover from peripheral neuropathy after discontinuation of oxaliplatin in 10% of patients [10]. Although co-administration of exenatide could not inhibit the incidence of oxaliplatin-induced mechanical allodynia, it facilitated recovery from oxaliplatin-induced neuropathy. Therefore, exenatide may be useful for the treatment of oxaliplatin-induced chronic peripheral neuropathy.

We also observed that oxaliplatin caused atrophy of myelinated fibers in the rat sciatic nerve on day 28. However, co-administration of exenatide had no effect on these changes, suggesting that exenatide cannot protect against oxaliplatin-induced axonal degeneration. On the other hand, co-administration of exenatide repaired oxaliplatin-induced axonal degeneration on days 42 and 63. With respect to pain behavior, exenatide relieved oxaliplatin-induced mechanical allodynia. Therefore, these reparations of axonal degeneration by exenatide may partially contribute to the reversal of oxaliplatin-induced mechanical allodynia. The present results support the involvement of axonal degeneration in the incidence of mechanical allodynia.

Oxaliplatin has been reported to inhibit neurite outgrowth in cultured PC12 and rat DRG cells and decrease cell survival in DRG cells [13, 21]. In the present study, oxaliplatin shortened the length of neurites and decreased cell viability of cultured PC12 cells. We also found that exenatide reversed oxaliplatin-induced neurite shortening in cultured PC12 cells. However, exenatide had no effect on the oxaliplatin-induced decrease in cell viability. These results suggest that exenatide ameliorates neurodegeneration but not cell death induced by oxaliplatin. This hypothesis is supported by histological results from the tissue of rats’ co-administered oxaliplatin and exenatide. Thus, exenatide appears to facilitate recovery from oxaliplatin-induced nerve injury. Recently, exendin-4, a GLP-1 receptor agonist, has been reported to facilitate the nerve regeneration after the crush nerve injury [30]. GLP-1 receptor is localized on large and small peptidergic neurons of adult rat DRG cells [42], suggesting the involvement of GLP-1 receptor in both the large and small sensory fiber functions. Taken together, these findings indicate that GLP-1 receptor may be new target of therapeutic agents of the nerve regeneration after chemotherapy-induced neuropathy.

GLP-1 receptor agonists have been reported to exhibit neuroprotective effects via increasing cAMP [43], having anti-apoptotic properties [44], or activating the c-Jun N-terminal kinase (JNK)/extracellular signal-regulated kinase (ERK) signaling pathway [45]. Additionally, GLP-1 receptor agonists can promote NGF-mediated differentiation in PC12 cells [46]. Furthermore, NGF has been reported to protect DRG neurons from oxaliplatin by modulating stress-activated protein kinases/JNK and ERK1/2. [47]. Recently, exendin-4 has been reported to enhance neurite outgrowth and neuronal survival through the activation of phosphatidylinositol-3'-phosphate kinase (PI3K) signaling pathway in cultured adult rat DRG neurons and PC 12 cells [42]. These molecular mechanisms may be involved in the effect of exenatide on oxaliplatin-induced mechanical allodynia.

GLP-1 receptors are specifically expressed on microglial cells in the spinal dorsal horn, and profoundly upregulated after peripheral nerve injury [48]. In addition, intrathecal injection of GLP-1 receptor agonists GLP-1 and exenatide potently alleviate formalin-, peripheral nerve injury-, bone cancer-, and diabetes-induced hypersensitivity in rats [48]. The anti-hypersensitive effects of GLP-1 receptor agonists are completely inhibited by not only GLP-1 receptor antagonism and gene knockdown but also the microglial inhibitor minocycline [48]. In an animal model of Parkinson’s disease, exendin-4 protects dopaminergic neurons by inhibition of microglial activation [49]. These findings suggest that microglia is involved in the anti-hypersensitive and neuroprotective effects of GLP-1 receptor agonists. On the other hand, the activation of spinal astrocytes but not microglia has been reported to be involved in the oxaliplatin-induced neuropathic pain [15–17]. In the present study, our data showed that exenatide could not inhibit the incidence of oxaliplatin-induced mechanical allodynia. Taken together, glial function might not be involved in the effect of exenatide in this study.

The present results also show that exenatide had no effect on oxaliplatin-induced tumor cytotoxicity in C-26 cells. Moreover, extended-release exenatide had no effect on the anti-tumor effect of oxaliplatin in tumor cell-implanted mice. Therefore, it is unlikely that exenatide influences the anti-tumor effect of oxaliplatin.

## Conclusions

The present results demonstrate for the first time that exenatide facilitates recovery from oxaliplatin-induced neuropathy without affecting anti-tumor activity. Some studies indicate that 10–20% of patients treated with oxaliplatin have type 2 diabetes [50, 51]. Therefore, exenatide may be useful for the treatment of oxaliplatin-induced peripheral neuropathy in colorectal cancer patients with type 2 diabetes.
